# Graphical Abstract in Scientific Research

**DOI:** 10.7759/cureus.45762

**Published:** 2023-09-22

**Authors:** Madhan Jeyaraman, Harish V K Ratna, Naveen Jeyaraman, Nicola Maffulli, Filippo Migliorini, Arulkumar Nallakumarasamy, Sankalp Yadav

**Affiliations:** 1 Orthopedics, ACS Medical College and Hospital, Dr MGR Educational and Research Institute, Chennai, IND; 2 Orthopedics, Rathimed Speciality Hospital, Chennai, IND; 3 Medicine, Surgery and Dentistry, University of Salerno, Baronissi, ITA; 4 Orthopadic, Trauma, and Reconstructive Surgery, Rheinisch-Westfälische Technische Hochschule (RWTH) University Medical Centre, Aachen, DEU; 5 School of Pharmacy and Bioengineering, Keele University Faculty of Medicine, Stoke-on-Trent, GBR; 6 Centre for Sports and Exercise Medicine, Barts and the London School of Medicine and Dentistry, Mile End Hospital, Queen Mary University of London, London, GBR; 7 Medicine, Shri Madan Lal Khurana Chest Clinic, New Delhi, IND

**Keywords:** visual abstract, research, social media, graphical abstract, graphics

## Abstract

A graphical abstract (GA) summarizes the key and important findings of an article graphically, potentially stimulating researchers to view the published manuscript. A GA should enhance dissemination, augment engagement, and impact clinical practice. Infographics play a key role in a quicker understanding of the significant findings of a manuscript. Few level 1 studies reported that GAs enhanced the engagement of readers on social media when compared to plain text abstracts. With the evolution of Industry 4.0, 5.0, and 6.0, GA plays a major role in understanding the technical aspects of various technologies. This article outlines tips to prepare an effective GA and reports the impact of GAs on research and clinical translation.

## Introduction and background

A graphical abstract (GA) is a graphic and visual summary of the important findings of a scientific article [1]. A GA should enhance a reader's ability to remember and recall information [2]. Recent research shows an eight-fold increase in the sharing of a visual abstract on social media in comparison to text-only abstracts; this has led to three times more visits by authors to the same article on the websites of journals [3]. In comparison to text abstracts, GAs have the upper hand: it takes about 6 seconds for an average reader to read about 20 words, whereas the same meaning could be understood within 1/4th of a second from a visual symbol [4].

Various social platforms, such as Facebook, Twitter, and Instagram, influence the lives of the general public and have become essential channels for communication among laypeople [5]. Among the total number of people using social media platforms, 1% are creators of content, 9% are editors, and the remaining 90% act as consumers of the content or seekers of the information provided to them [6,7]. In the present digital era, where there is exponential growth in the field of orthopedic research, GA plays a major role in helping readers preview the study content and decide whether to read the entire article [1,8,9].

## Review

GA enhances the dissemination of research among the readers, augments the engagement of research scholars, and creates a profound impact on clinical practice, which helps in clinical translation [1]. GA provides a bird's-eye view of the article, making it more attractive and appealing to readers, entices them to have an in-depth look, and makes it easier for the researcher to share or re-post on social media to engage like-minded individuals [10].

Triad of GA

**Figure 1:**
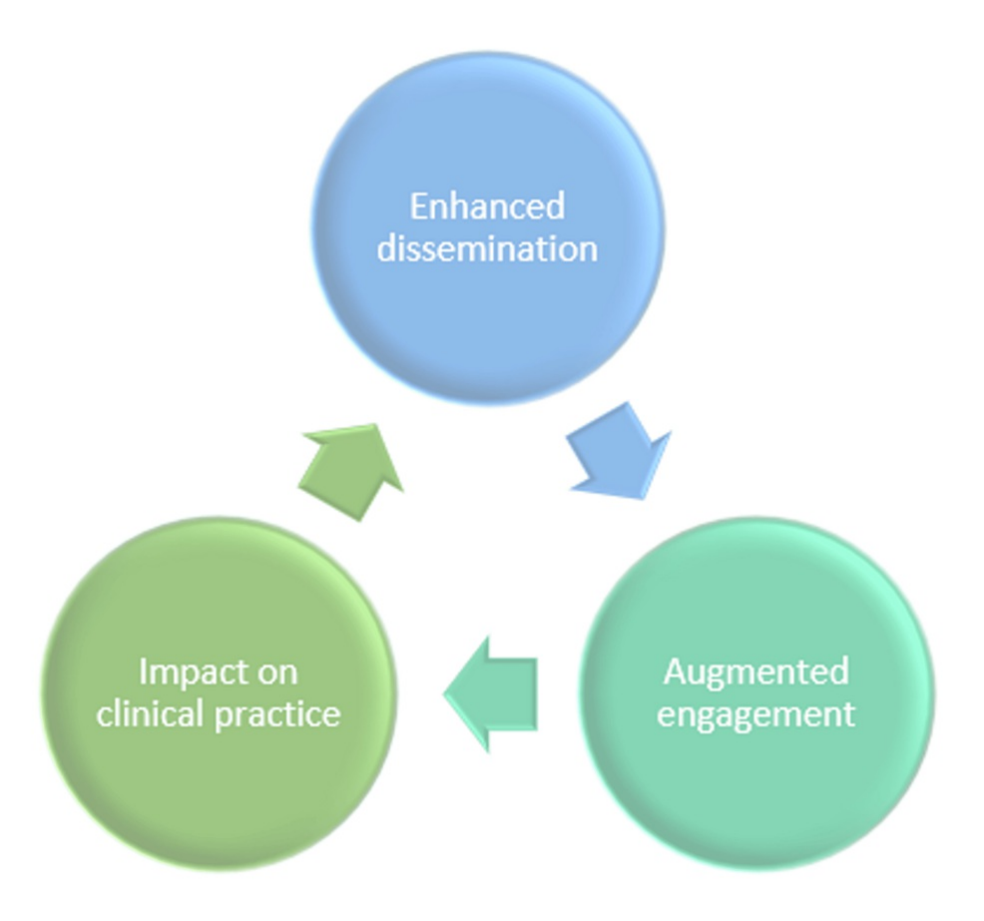
Triad of graphical abstract Picture courtesy of Dr. Madhan Jeyaraman

The triad of GA is depicted in Figure 1.

The triad of GA is a) enhanced dissemination [GAs enhance the dissemination of research-related articles]. In the natural evolution of scientific information communication, videos and graphics will generate higher "hits" in comparison to text-only abstracts. Visual abstracts (VAs) increase the dissemination of research on social media by eight-fold compared to text-only abstracts [1]], b) augmented engagement [GAs provide a framework for increased engagement of like-minded researchers on social platforms. GAs act as a reminder to the reader and help in discussing the contents of the article in a forum], and c) impact on clinical practice [This may be difficult to evaluate, but, for example, the article published in "Annals of Surgery" had a great impact on disseminating information about antibiotic stewardship]. Producing an effective GA necessitated distilling the core research points into 2-3 eye-catching sentences to pique readers' interest . The prototype structure of GA is depicted in the graphical abstract of the manuscript. A GA can be divided into four areas, namely: a) the title area, b) the methods and cohort area, c) the findings area, and d) the conclusion area (Table 1).

**Table 1:** Areas of graphical abstract

Areas	Significance
A – Title	Depicts the context of the research study
B – Methods and cohort	Type of research study to be mentioned Study time frame and follow-up period to be mentioned
C – Findings	Highlight the validity of research findings Interpretation of findings by the readers
D – Conclusion	Summarize the key points of the manuscript Intended to highlight the primary outcome of the study Must mention take-home message

**Figure 2:**
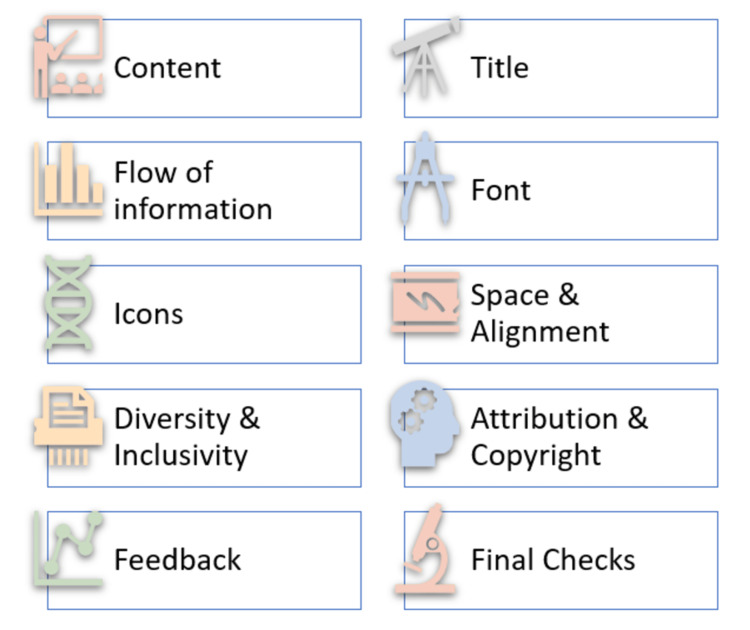
Tips for creating an effective graphical abstract Picture courtesy of Dr. Madhan Jeyaraman

The tips and tricks for creating an effective GA are reported in Figure 2.

**Figure 3:**
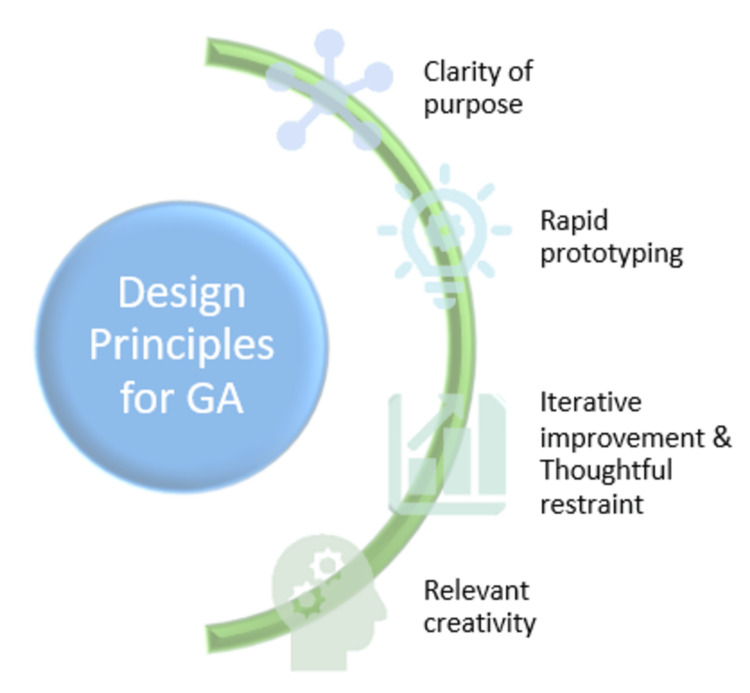
Design principles for a good graphical abstract Picture courtesy of Dr. Madhan Jeyaraman

The design principles for a good GA are shown in Figure 3.

The elements of GA are: a) display of the title, name of the author, and name of the journal at the top of the GA; b) display of the journal’s logo at the bottom of the GA; c) display of the graphical presentation of research results; and d) display of the take-home message.

In general, GAs represent the face value of the underlying research article and are presented at the beginning of the article, after the text-only abstract. GAs provide a summary of the research article in the form of visual icons in single or dual-colored tones.

Resources to prepare GA

A social media platform enables the sharing of different GAs with various styles and structures. Peer-reviewed journals have started to provide guidelines for GAs based on recently published evidence, which would help in standardizing the outputs and would ensure the consistency and validity of the GAs published in a given journal. Many sources are available to provide guidelines for preparing a GA, which are as follows: a) Visual Abstract Primer (edited by Andrew Ibrahim), which covers topics such as creating a visual abstract and leveraging a visual abstract for dissemination [11], b) Andrew Ibrahim’s Guidelines to Standardize GAs for Scientific Research [1], and c) Michelle Lim’s short course on designing and the design process of GAs [12].

To prepare an informative GA effectively, one should be able to compile the research-related information and be able to reproduce it understandably under three main sections, namely a) the methods of the study, b) the important findings of the study, and c) the conclusions of the research study. There is no need for costly and complex illustrative software or additional graphical or artistic skills. The only additional need is good creativity to figure out the way to represent the findings in the form of visual icons, as well as the capacity to compile the information into small sections [12].

Process of creating and disseminating GA

**Figure 4:**
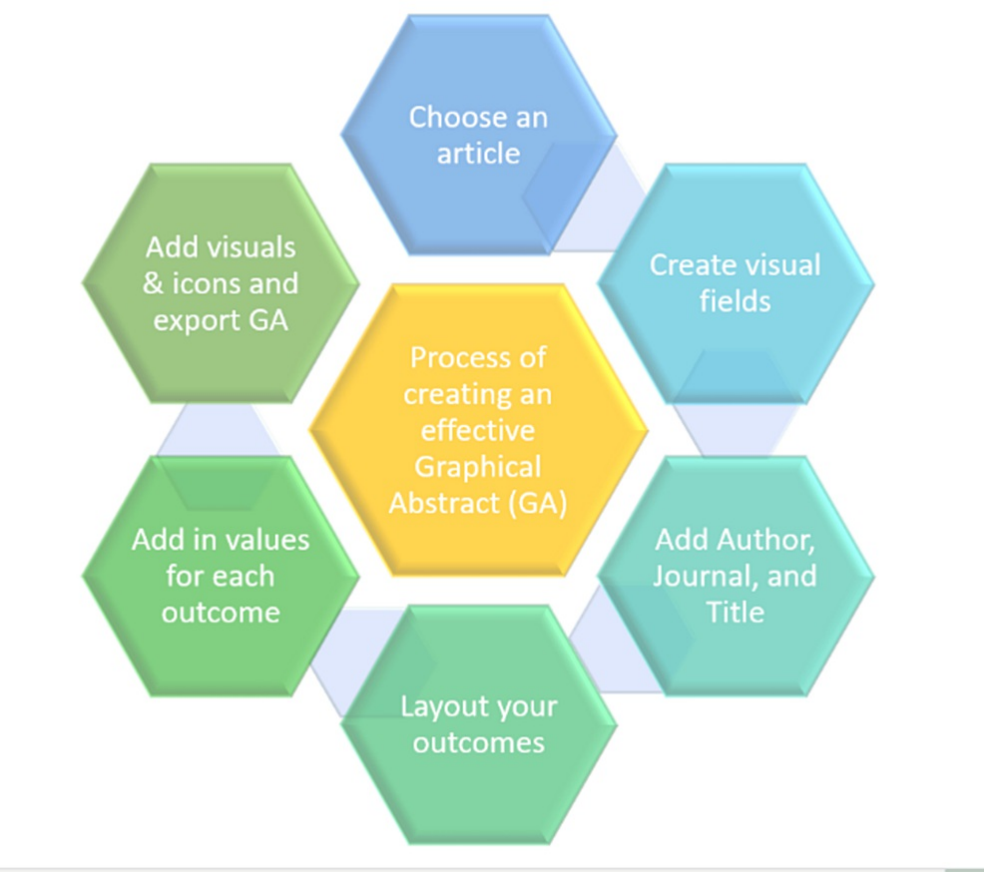
Process of creating an effective graphical abstract Picture courtesy of Dr. Madhan Jeyaraman

The process of creating an effective GA is depicted in Figure 4.

Recently, more than 50 top journals, including "The New England Journal of Medicine", "Clinical Spine Surgery", and "JAMA", have adopted GAs to disseminate research through various social media platforms, particularly Twitter. By disseminating GAs through social media, over some time, the number of citations for the manuscript and the journal’s impact factor also steadily increased.

A few questions about GAs are being debated, namely: a) who can create GA, the author or the journal’s editorial team?; b) what are the specifications for creating GA to be included in the instruction for the author's section; c) who will review the GA (the author, the reviewer, the section editor, the editor-in-chief); d) who, how, and where will GAs be disseminated?; and e) if a journal has the answer to these questions, the dissemination of GAs among research scholars and clinicians will improve the quality of the GAs and maximize the impact of the research.

Cross-talk between GA and social media

In the recent past, there has been active participation by patients in surgical research, but their access to the results of the study in which they took part is limited to the commentary of non-expert individuals [13,14]. Research scientists must deliver the results of the research in a format easily accessible to readers. Various initiatives have been developed to enhance this communication and eradicate the communication gap, including the National Institute of Health Research's (NIHR) 'Make It Clear" campaign [15] and the British Medical Journal's ‘Patient and Public Partnership’ initiative [16]. Various social media platforms have become popular over the last 3-5 years to disseminate surgical research-related work [17]. This has been witnessed in growing instances, such as conference-related and specialty-related hashtags, facilitating discussion in international forums and journal-specific journal clubs, facilitating post-publication discussion among peers, and discussing new techniques of surgery through live surgical videos [17,18]. Another new initiative is GAs, which have helped to disseminate the results of surgical research to an academic audience [3,19].

GA can make an article more pleasant and understandable when compared to text-only tweets, allowing easy and fast recall of the important points of the article relevant to the research scholar [20]. The impact of GAs may rise or fall depending on the number of followers of the individual’s social platform account or may differ based on the particular specialty, subsequent sub-specialties, and social media platforms. The mean number of engagements on viewing visual abstracts (VAs) was significantly higher at 7 and 30 days than plain English texts, whereas the crossover results were similar for orthopedic research on social platforms, but greater overall public engagement was observed with visual abstracts than plain text tweets [20]. Some level 1 studies depict the usage of VAs through social media [3,21,22], with the strongest correlation between VAs and the dissemination of research on social media [3]. VAs may not increase the number of reads and downloads of the fully published manuscript [23]. These GAs make the article more appealing for the readers to grasp the main crux of that particular manuscript.

Challenges and pitfalls of GA

Any form of medium to exchange scientific research among readers has various challenges and pitfalls. Given the emergence of GA in various journals, the results of the underlying scientific research will be oversimplified, which may result in a misinterpretation of the results. When the readers systematically use GAs as a substitute for the whole manuscript, and this is not read, they may eventually lose the capability to evaluate and appraise manuscripts. When a GA is produced by a third party, the output of the GA may not be reviewed by the author and their team, which may theoretically introduce fallacies, misinterpretations, biases, or inaccuracies. The possibility of salami slicing may happen when the creation of GA is outsourced. The quality control of GAs remains a challenge when their production is outsourced. Though GA appears simple and lucid in understanding by the readers, the third party does not necessarily understand the subject to be able to convey the right message. The major setback for GA is the availability of space in the given area. So, authors tend to report only the positive findings of the study [12], inevitably introducing biases. GAs must be submitted with the main manuscript and undergo peer review.

Future directives

Future studies should focus on the impact of GAs on the citation of a scientific article. Among the available social media platforms, the best and ideal platform must be identified for promoting and propagating research among various orthopedic researchers. The journal may want to establish GA creation teams that have to undergo training in infographics, and proper feedback has to be obtained for sufficient training to produce GAs. The Nephrology Social Media Collective is a successful example of how to promote and propagate research through the creation of GA, podcasts, blogs, newsletters, and research games [24,25]. When the journal’s editorial team produces GAs, misinterpretation and bias, have to be minimized. However, the journal has to issue the guidelines and specifications for preparing GA to avoid the hassle in the editorial workflow. The creation of a GA repository within the journal will encourage readers to refer to the research results when needed [26]. The available way to search GA is through the hashtags #visualabstract or #graphicalabstract. GAs must have a link in the manuscript when searched via standard indexing and abstracting databases. An alternate form for searching GA is the creation of a GA repository after obtaining permission from the native journal for sharing and disseminating the GA.

**Figure 5:**
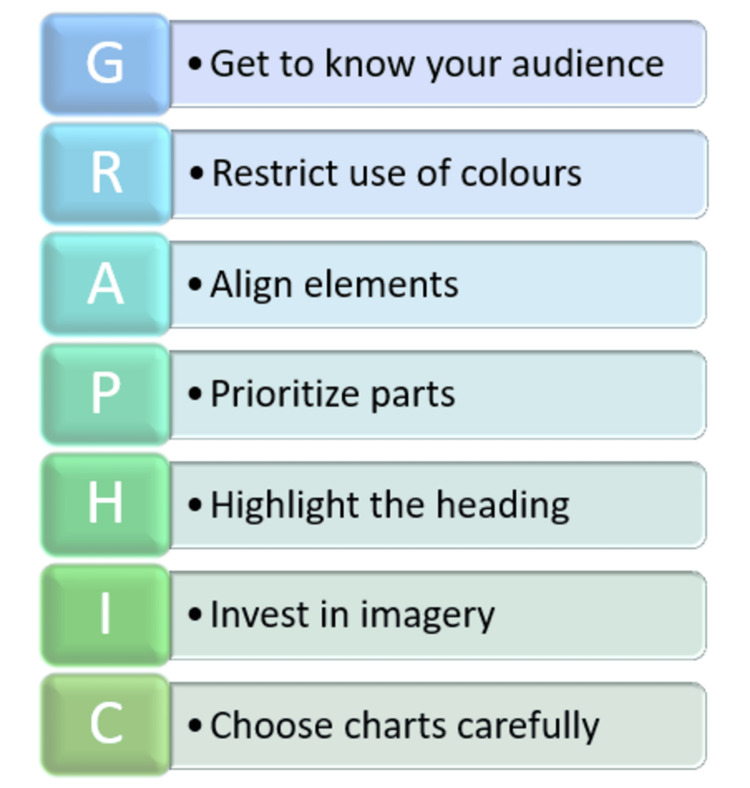
A 'GRAPHIC' model for an effective graphical abstract Picture courtesy of Dr. Madhan Jeyaraman

GA can be presented at scientific conferences as a poster by using infographics and icons. This may attract more attention among conference attendees for the key findings of the research. GA can be presented as oral presentations in various forums, including clinical lectures, departmental and academic meetings, or case discussions, to project the research data more clearly. Finally, GA increases the visibility of the speaker and the research among like-minded research scholars. The GRAPHIC model must be adhered to to form a sound and effective GA, as depicted in Figure 5.

**Figure 6:**
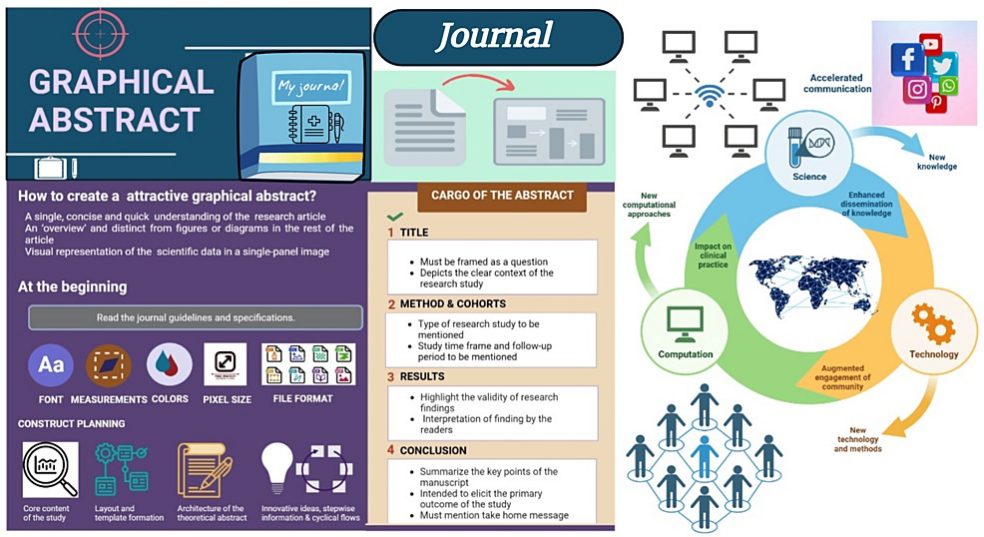
Schematic presentation of a graphical abstract Picture courtesy of Dr. Madhan Jeyaraman

A schematic presentation of GA is depicted in Figure 6.

## Conclusions

The emergence and integration of GAs into the landscape of scientific communication signifies an evolution in how research is presented and understood in our digital age. GAs, with their visual appeal and concise representation, are bridging the gap between dense scientific content and its audience, both experts and laypeople. Their popularity on social media platforms underscores the shift towards a more visual and immediate form of information consumption. However, like all innovations, GAs come with challenges, including potential misinterpretation and concerns about oversimplification. As we look forward, it's imperative for the scientific community to refine guidelines for creating and reviewing GAs, ensuring they both accurately represent the research and remain engaging to the audience. By doing so, we can harness the potential of GAs to improve the dissemination, engagement, and impact of scientific research without compromising the integrity of the content. This era of visual communication in science, championed by GAs, underscores the adage: a picture, indeed, might be worth a thousand words.
